# Nightingale Nursing Home, Derby

**Published:** 1905-08-19

**Authors:** 


### NIGHTINGALE NURSING HOME, DERBY.

This building was formerly known as Chetwynd House, and it occupies the corner site of London Road and Trinity Street. It is, of course, practically impossible to convert a private dwelling house into a really satisfactory hospital, and except on the score of economy such conversion is a step rarely to be advised. The architect has a troublesome task allotted to him, and, unless large additions are made, all that can be done is to get rid of as many defects as possible; and of these defects the absence of cross-ventilation of the rooms is the one which is most likely to be well-nigh impossible to banish. It is just this point which is considered to be all-important in a modern hospital; therefore we advise every board of managers, where funds are likely to be forthcoming, not to have anything to do with an existing building unless such building can be kept as an administrative department and have new wards added.

On the ground floor of this Home there is to the left, on entering from Trinity Street, the matron's sitting-room, and at the end of the hall is the door of No. 1 Ward. This ward is about 22 feet long and 17 feet wide, and it has, in addition, two large bay windows, one being in the side of the room and the other in the end, so that there will be in this ward a fair amount of cross-ventilation and plenty of light. Neither here nor in any other ward do the plans state how many beds will be placed therein; but assuming a ceiling height of 12 feet, ward No. 1 will contain approximately 5,200 cubic feet of space, and therefore would be suitable for three or four beds. The room contains a fixed lavatory basin, an open fire-place, and a hot-water radiator. The main staircase is placed in the hall between No. 1 Ward and No. 2 Ward, and the latter has three windows all in the side facing the garden. There is one door opening from the hall and another leading to the passage connecting this ward with No. 3 Ward. The passage also gives access to the utensil room, sinks, and closet; and the latter are not cut off from the main by a cross-ventilated passage.

The kitchen is placed at the back of No. 2 Ward, and there is a conveniently placed dining-room, a receiving-room, a waiting-room, a dispensary, and a laundry.

The first floor contains Wards No. 4 and No. 5, six bedrooms, and a bath-room.

At the end of the laundry yard is a coach-house, and over this is the isolation ward with its lavatory, closet, and nurse's room; all being well arranged.

The architects are Messrs. Wright and Thorpe, of Derby. The cost is not stated. We regret this, as if the total sum paid for the building and for having it altered were known, it would be possible to form some idea of the difference in cost between it and an entirely new nursing home.

